# EventHD: Robust and efficient hyperdimensional learning with neuromorphic sensor

**DOI:** 10.3389/fnins.2022.858329

**Published:** 2022-07-27

**Authors:** Zhuowen Zou, Haleh Alimohamadi, Yeseong Kim, M. Hassan Najafi, Narayan Srinivasa, Mohsen Imani

**Affiliations:** ^1^Department of Computer Science, University of California, Irvine, Irvine, CA, United States; ^2^School of Engineering, University of California, Los Angeles, Los Angeles, CA, United States; ^3^Daegu Gyeongbuk Institute of Science and Technology, Daegu, South Korea; ^4^School of Computing and Informatics, University of Louisiana, Lafayette, LA, United States; ^5^Intel Labs, Santa Clara, CA, United States

**Keywords:** hyperdimensional computing, neuromorphic sensor, brain-inspired computing, Dynamic Vision Sensor, machine learning

## Abstract

Brain-inspired computing models have shown great potential to outperform today's deep learning solutions in terms of robustness and energy efficiency. Particularly, Hyper-Dimensional Computing (HDC) has shown promising results in enabling efficient and robust cognitive learning. In this study, we exploit HDC as an alternative computational model that mimics important brain functionalities toward high-efficiency and noise-tolerant neuromorphic computing. We present EventHD, an end-to-end learning framework based on HDC for robust, efficient learning from neuromorphic sensors. We first introduce a spatial and temporal encoding scheme to map event-based neuromorphic data into high-dimensional space. Then, we leverage HDC mathematics to support learning and cognitive tasks over encoded data, such as information association and memorization. EventHD also provides a notion of confidence for each prediction, thus enabling self-learning from unlabeled data. We evaluate EventHD efficiency over data collected from Dynamic Vision Sensor (DVS) sensors. Our results indicate that EventHD can provide online learning and cognitive support while operating over raw DVS data without using the costly preprocessing step. In terms of efficiency, EventHD provides 14.2× faster and 19.8× higher energy efficiency than state-of-the-art learning algorithms while improving the computational robustness by 5.9×.

## 1. Introduction

Many applications run machine learning algorithms to assimilate the data collected in the swarm of devices on the Internet of Things (IoT). Sending all the data to the cloud for processing is not scalable and cannot guarantee a real-time response. However, the high computational complexity and memory requirement of existing machine learning models hinder usability in a wide variety of real-life embedded applications where the device resources and power budget is limited (Denil et al., 2013; Zaslavsky et al., 2013; Sun et al., 2016; Xiang and Kim, 2019). Therefore, we need alternative learning methods to train less-powerful IoT devices while ensuring robustness and generalization.

System efficiency comes from sensing and data processing. Unlike classical vision systems, neuromorphic systems try to efficiently capture a notion of seeing motion. Although bio-inspired learning methods, i.e., spiking neural networks (SNNs) (Schemmel et al., 2006; Liu et al., 2014), address issues related to energy efficiency (Huh and Sejnowski, 2017; Neftci et al., 2019), these systems still require to provide robustness and brain-like cognitive support. For example, the existing bio-inspired method cannot integrate perceptions and actions.

To achieve real-time performance with high energy efficiency and robustness, our approach redesigns learning algorithms using strategies that closely model *the human brain* at an abstract level. We exploit Hyper-Dimensional Computing (HDC) as an alternative computational model that mimics important brain functionalities toward high-efficiency and noise-tolerant computation (Kanerva, 2009; Rahimi et al., 2016b; Pale et al., 2021, 2022; Zou et al., 2021). HDC supports operators that emulate the behavior of associative memory and enables higher cognitive functionalities (Gayler, 2004; Kanerva, 2009; Poduval et al., 2022). In HDC, objects are thereby encoded with high-dimensional vectors, called *hypervectors*, which have thousands of elements (Kanerva, 2009; Rahimi et al., 2016b; Imani et al., 2019c). HDC incorporates learning capability along with typical memory functions of storing/loading information. HDC is well suited to enable efficient and robust learning because: (i) HDC models are computationally efficient to train, highly parallel at heart, and amenable to hardware-level optimization (Wu et al., 2018; Imani et al., 2019b), (ii) HDC supports single-pass learning tasks using a small amount of data (Rahimi et al., 2016a), and (iii) HDC exploits redundant and holographic representation with significant robustness to noise and failure in hardware (Li et al., 2016).

There are a few recent studies that tried to exploit HDC to process neuromorphic sensors (Mitrokhin et al., 2019; Hersche et al., 2020). However, these solutions are not end-to-end as they operate over preprocessed data. Pre-processing is a costly *time-image* feature extraction that maps noisy neuromorphic data to a small number of features. This preprocessing has the following drawbacks: (1) dominates the entire computation cost (Mitrokhin et al., 2019), (2) reduces the necessity of using HDC-based learning, as a less-sophisticated learning algorithm can also provide acceptable accuracy over-extracted features, (3) requires heterogeneous data processing and non-uniform data flow to accelerate preprocessing and HDC-based steps, and (4) finally, suffers from low computational robustness, as the preprocessing step operates over original data with high sensitivity to noise (Hersche et al., 2020; Imani et al., 2020).

In this article, we proposed EventHD, a neurally-inspired hyperdimensional system for real-time learning from a neuromorphic sensor. To the best of our knowledge, EventHD is the first HDC-based algorithm that provides robust and efficient learning by operating over raw spike data from a neuromorphic sensor. The main contributions of the article are listed as follows:

- We propose a novel hyperdimensional encoding module that receives neuromorphic data and maps it to holographic hyperdimensional spikes with highly sparse representation. Our encoding preserves the spatial and temporal correlation between the input events to naturally keep their similarity in high dimensions. In addition, our encoding module preserves asynchrony from the neuromorphic devices.
- We enable supervised and semi-supervised learning using HDC-based algorithms. Our solution enables single-pass training where the HDC model can be updated in real-time by one-time looking at each train data. EventHD also defines confidence for each prediction and enables self-learning from unlabeled data.
- We show EventHD capability to memorize associated perception-action and define the theoretical capacity of this model to reason based on prior knowledge.

We evaluate EventHD efficiency and accuracy over various data collected from DVS sensors. Our results indicate that EventHD can provide real-time learning and cognitive support while operating over raw DVS data without using the costly preprocessing step. Furthermore, EventHD in a single node provides 14.2× and 19.8× faster and higher energy efficiency than state-of-the-art learning algorithms while improving the computational robustness by 5.9×.

## 2. Preliminary and overview

### 2.1. Hyperdimensional learning

The brain's circuits are massive in terms of numbers of neurons and synapses, suggesting that large circuits are fundamental to the brain's computing. Hyperdimensional computing (HDC) (Kanerva, 2009) explores this idea by looking at computing with ultra-wide words—i.e., with very high-dimensional vectors or hypervectors. The fundamental computation units in HDC are high dimensional representations of data known as “hypervectors” constructed from raw signals using an encoding procedure. There exist a huge number of different, nearly orthogonal hypervectors with the dimensionality in the thousands (Kanerva, 1998). This lets us combine such hypervectors into a new hypervector using well-defined vector space operations while keeping the information of the two with high probability. Hypervectors are holographic, that is, the information encoded into the hypervector is distributed “equally” over all the components. In our case, it is done using (pseudo)random hypervectors with i.i.d. components as our ingredients for the encoding. A hypervector contains all the information combined and spread across all its components in a full holistic representation so that no component is more responsible for storing any piece of information than another.

In HDC, hypervectors are compositional—they enable computation in superposition, unlike standard neural representations (Kanerva, 2009). These HDC operations allow us to reason about and search through images that satisfy pre-specified constraints. These composite representations can be combined using HDC operations to encode temporal information or complex hierarchical relationships. This capability is especially powerful for understanding the relationship between objects in images in both time and space. These operations are simple in HDC and require only trivial element-wise arithmetic. By contrast, to achieve the same effect in a neural network, e.g., spiking neural networks (Wang et al., 2018), we would need to assign images corresponding to composite classes a new label and train a separate model for prediction. HDC also provides a natural way to preserve temporal information using a permutation operator (Rahimi et al., 2016b). For example, we encode a sequence of video frames while preserving the temporal structure. This would allow us to efficiently compute a similarity score for entire sequences of video using a standard HDC similarity search, which is extremely efficient in hardware (Li et al., 2016).

### 2.2. Hyperdimensional primitives

Let us assume ${\vec{H}}_{1}$, ${\vec{H}}_{2}$ are two randomly generated hypervectors ($\vec{H}\in {\left\{-1,+1\right\}}^{D}$) and $\delta \left({\vec{H}}_{1},{\vec{H}}_{2}\right)≃0$, where δ is the cosine similarity function, $\delta \left({\vec{H}}_{1},{\vec{H}}_{2}\right)=\frac{{\vec{H}}_{2}\cdot {\vec{H}}_{2}}{∥{\vec{H}}_{1}∥\cdot ∥{\vec{H}}_{2}∥}$.

Binding (*) of two hypervectors ${\vec{H}}_{1}$ and ${\vec{H}}_{2}$ is done by component-wise multiplication (XOR in binary) and denoted as ${\vec{H}}_{1}*{\vec{H}}_{2}$. The result of the operation is a new hypervector that is dissimilar to its constituent vectors i.e., $\delta \left({\vec{H}}_{1}*{\vec{H}}_{2},{\vec{H}}_{1}\right)\approx 0$; thus, binding is well suited for associating two hypervectors. Binding is used for variable-value association and, more generally, for mapping.

**Bundling (+)** operation is done *via* component-wise addition of hypervectors, denoted as ${\vec{H}}_{1}+{\vec{H}}_{2}$. The bundling is a memorization function that keeps the information of input data in a bundled vector. The bundled hypervectors preserve similarity to their component hypervectors i.e., $\delta \left({\vec{H}}_{1}+{\vec{H}}_{2},{\vec{H}}_{1}\right)>>0$. Hence, a bundling of hypervectors is well suited for representing the set of elements corresponding to the hypervectors that are bundled, and we may test their membership by a similarity check.

**Permutation (**ρ**)** operation, $\rho^{n}\left(\vec{H}\right)$, shuffles components of $\vec{H}$ with *n*-bit(s) rotation. The intriguing property of the permutation is that it creates a near-orthogonal and *reversible* hypervector to $\vec{H}$, i.e., $\delta \left(\rho^{n}\left(\vec{H}\right),\vec{H}\right)≃0$ when *n* ≠ 0 and $\rho^{-n}\left(\rho^{n}\left(\vec{H}\right)\right)=\vec{H}$. Thus, we can use it to represent *sequences* and *orders*.

**Reasoning** is done by measuring the similarity of hypervectors. We design the encoding of the hypervectors such that the similarity between the hypervectors reflects the similarity between the entities that they represent.

### 2.3. Overview

This article focuses on learning over data collected by the Dynamic Vision Sensor (DVS). Unlike a normal camera that captures data synchronously and frame-based, a DVS camera mimics the mechanics of the human retina by detecting and recording the changes in the illumination of a pixel asynchronously, sending a stream of events to the memory. This leads to sparse data because only a small subset of pixels reports events at any time, with rich temporal information, because of the asynchrony, rendering it much more difficult to train. Nevertheless, DVS data has been actively studied and researched in the context of neuromorphic computing, e.g., in conjunction with the Spiking Neural Network, for various image-related tasks such as gesture recognition and object classification (Massa et al., 2020).

In this article, we present EventHD, an end-to-end framework for robust, efficient hyperdimensional learning from the neuromorphic sensor. Unlike all prior works that operate over preprocessed data, to the best of our knowledge, EventHD is the first HDC-based solution that directly operates over raw neuromorphic data. We first develop a novel hyperdimensional encoding scheme to map event-based neuromorphic data into high-dimensional space. EventHD exploits hyperdimensional mathematics to preserve spatial and temporal information from raw sensor data (Section 3). Next, we introduce novel algorithm solutions to perform classification and self-learning over the encoded data (Section 4). This includes enabling single-pass classification and supporting association and memorization over perception-action space (Section 5).

## 3. EventHD spatial encoding

We exploit hyperdimensional computing mathematics to design a novel encoding module that receives event-based spiking data and generates high-dimensional data. Our HDC mapping is not a random projection. Instead, it preserves the temporal and spatial correlation between the input data. The goal of this encoder is to represent spikes in a holographic representation; thus, a single noisy spike in original data represents a pattern of neural activity in high-dimensional space. The holographic representation means that the information of each original spike will be uniformly distributed over all dimensional of our encoded hypervector. In addition, given that our encoding is purely event-based, it can also be operated in an asynchronous setting, reacting to DVS events, thus preserving asynchrony.

Let us assume the output of the DVS camera is in a form of *E*_*k*_ = (**x**_*k*_, *t*_*k*_, *p*_*k*_), signaling at time *t*_*k*_ and location **x**_*k*_ = (*x*_*k*_, *y*_*k*_). When the illumination change surpasses a threshold *p*_*k*_·*C*, where *p*_*k*_ ∈ {−1, 1} and *C* is a predetermined threshold. For simplicity, we first explain how our encoder preserves the spatial correlation of spikes in holographic high-dimensional space. Then, we add temporal locality as a memorization term to our encoder.

### 3.1. Base generation

**Figure 1:**
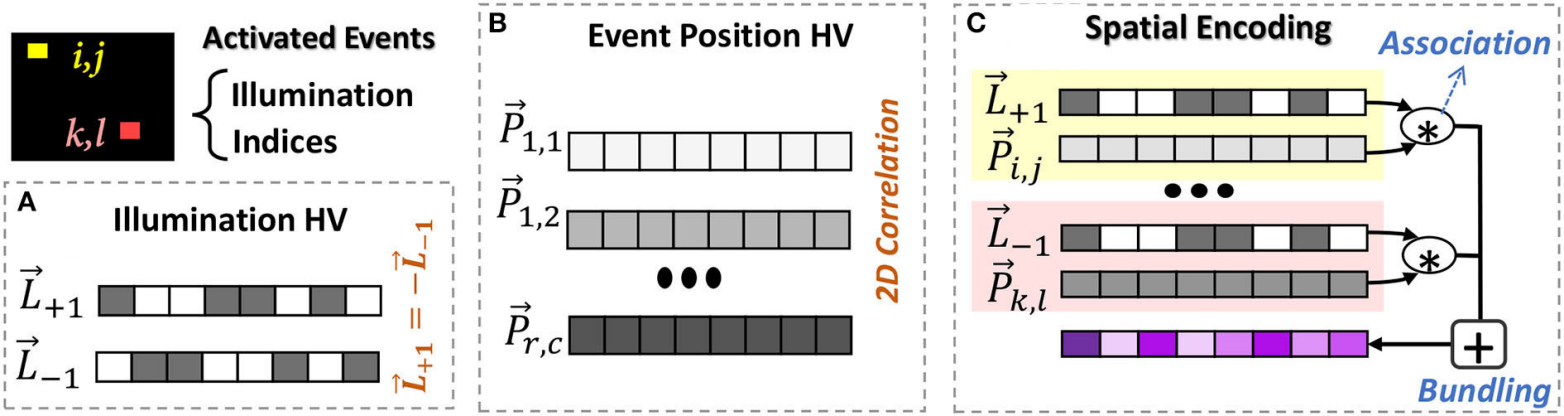
EventHD spatial encoding: **(A)** base generation for illumination, **(B)** base generation for event position to keep 2D correlation of pixels in neuromorphic image, and **(C)** spatial encoding that associates and memorizes illumination and position hypervectors.

Hyperdimensional computing encoding is performed based on a set of base or seed hypervectors. The base hypervectors represent the basic alphabet of the data. For an example of DVS data, the alphabets are illumination changes and the position of events. Figures 1A,B shows the EventHD base generation procedure.

- **Illumination hypervector:** The illumination change has two possibilities, increase or decrease. This information can be represented using a random hypervector, where ${\vec{L}}_{1}\in {\left\{-1,+1\right\}}^{D}$ and ${\vec{L}}_{-1}=-{\vec{L}}_{1}$ (Figure 1A).
- **Event-position hypervector:** The information of event positions can be represented using a set of position hypervectors $\left\{{\vec{P}}_{0,0},\cdots \;,{\vec{P}}_{r,c}\right\}$, where the indices represent the row and column location of an event in the input (*r* × *c* pixels in DVS camera). The position hypervectors can not be generated randomly, as they need to preserve the spatial correlation between the neighbor events (Figure 1B). In other words, the events with closer physical distance have a higher correlation. Using techniques introduced in Gallant and Culliton (2016) and Kim et al. (2021), we generate position hypervectors in three steps: (1) partition events into smaller non-overlapping *k* × *k* windows, and (2) generate randomly generated hypervectors for pixels located on the corner of windows. For example, we generate random hypervector for $\left\{{\vec{P}}_{0,0},{\vec{P}}_{0,k},{\vec{P}}_{k,0},{\vec{P}}_{k,k}\right\}$. This repeats over all *k* × *k* windows. Since these vectors are randomly chosen and they are in high-dimensional space, they are nearly orthogonal ($\delta \left({\vec{P}}_{0,0},{\vec{P}}_{k,k}\right)≃0$). (3) For all intermediate pixels, we perform interpolation to generate correlated hypervectors. Each pixel will get partial dimensions from the position hypervectors located in the four corners of a *k* × *k* window. The number of dimensions to take from each corner hypervectors depends on the relative position of a pixel within the window such that the generated position hypervectors preserve the 2D spatial correlation between events' positions. For an in-depth description of the spatial interpolation, readers are referred to Gallant and Culliton (2016) and Kim et al. (2021).

### 3.2. Spatial encoding

In a given time, our encoder looks at neuromorphic data as an image with few activated spikes/events. The goal of the encoder is to map this data into high-dimensional space using pre-generated base hypervectors. The encoding is performed in two steps, as shown in (Figure 1C):

**Associating event-illumination:** For every activated event, our encoder exploits a binding operation to associate each event position with the corresponding illumination hypervector. For example, if an event in position [*i, j*] is activated, our encoder associates information using: ${\vec{P}}_{i,j}\times {\vec{L}}_{i},j$, where ${\vec{L}}_{i},j$ can be ${\vec{L}}_{+1}$ or ${\vec{L}}_{-1}$ depending on illumination direction. The bound hypervector preserves position and illumination information in a new hypervector that is nearly orthogonal to its operands. We perform the same association for all activated events.

**Event memorization:** In HDC, bundling acts as memorization. We exploit this feature to memorize the information of all activated events in a given time. Our solution bundles associated hypervectors for all activated events: $\vec{S}=\sum_{i=1}^{r}\sum_{j=1}^{c}\left({\vec{P}}_{i,j}*{\vec{L}}_{i,j}\right)$ when (*r, c*) has a spike event. The memorization and summation only happens for pixels that have spike events.

### 3.3. EventHD temporal encoding

**Figure 2:**
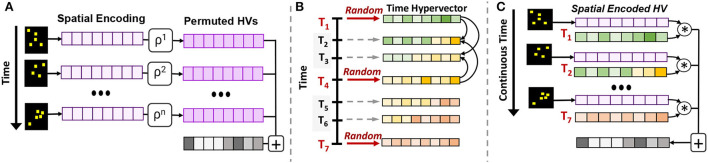
EventHD temporal encoding: **(A)** permutation-based encoding, **(B,C)** correlative time hypervector used for associated-based encoding.

Let us consider actual neuromorphic data with temporal spikes/events. As we explained in Section 3.2, for all events that happen in a time window, we exploit spatial encoding to map all events into single hypervectors. As time moves on, the information of new events needs to be encoded into a new hypervector. Figure 2 shows two solutions to memorizing signals and keeping temporal information.

**Permutation-based:** To incorporate a notion of time, our encoder represents the position of each time slot using a single permutation. The permutation in HDC is defined as a rotational shift, where the permuted hypervector is nearly orthogonal to its original vector. Figure 2A shows how *n* spatial-encoded data can be temporally combined through time. For example, to memorize three consecutive encoded signals, ${\vec{S}}_{1}$, ${\vec{S}}_{2}$, and ${\vec{S}}_{3}$, we encode them into a single hypervector by $\vec{H}=\rho^{1}\left({\vec{S}}_{1}\right)*\rho^{2}\left({\vec{S}}_{2}\right)*\rho^{3}\left({\vec{S}}_{3}\right)$, where binding (*) and permutation (ρ) memorize sensor value and position. This encoding preserves the temporal information of events. Although permutation can preserve sequence information, it is a very exclusive operation that loses the information of continuous-time. For example, even when two continuous events are identical, this temporal encoding is orthogonal ($\delta \left(\rho^{1}\vec{S},\rho^{2}\vec{S}\right)≃0$).

**Association-based:** To give a notion of continuous time, we exploit binding operations to keep temporal correlative information. Our temporal encoding is performed using the following steps: (1) Similar to spatial encoding, we generate a set of correlated hypervectors to preserve temporal correlation. As Figure 2B shows, our solution splits time into smaller *t*-size windows. We generate a random hypervector for each time that is a factor of *t*. For example, we generate random hypervectors representing $\left\{{\vec{T}}_{0},{\vec{T}}_{t},{\vec{T}}_{2t},\cdots \;,{\vec{T}}_{kt}\right\}$, where indices represent time steps. (2) We perform interpolation to generate a correlated hypervector representing intermediate times. Given time *t*_0_ ∈ [*jt*, (*j* + 1)*t*] for some non-negative integer *j*, *T*_*t*_0__ is generated by taking components from *T*_*jt*_ and *T*_(*j*+1)*t*_ in (*j* + 1)*t* − *t*_0_:*t*_0_− *jt* ratio such that the similarity between the three reflects their original correlation. For an example of *t* = 3, ${\vec{T}}_{1}$ will be 66.6% similar to ${\vec{T}}_{0}$ and 33.3% similar to ${\vec{T}}_{3}$. Our temporal correlation goes beyond a single-window; hypervectors in two neighbor windows are also correlated.

As Figure 2 shows, we exploit the time-base correlated hypervectors to preserve temporal information. Let us assume ${\vec{H}}_{i}$ is a hypervector of events happening in a time slot *i*. Our encoding preserves temporal correlation of *p* time-slot using: $H=\sum_{i=1}^{p}\left({\vec{T}}_{i}*{\vec{S}}_{i}\right)$.

## 4. EventHD classification

In this section, we introduce HDC-based classification algorithms that can directly learn from encoded query data. This includes developing algorithms that can effectively learn from both labeled and unlabeled data.

### 4.1. Supervised learning

EventHD supports two types of classification: *accumulative* and *adaptive* learning. Both methods are a single-pass approaches that can construct a learning model by one-time looking at training data. The single-pass model is significantly fast and efficient and enables learning from the data stream with no need for off-chip memory.

**Figure 3:**
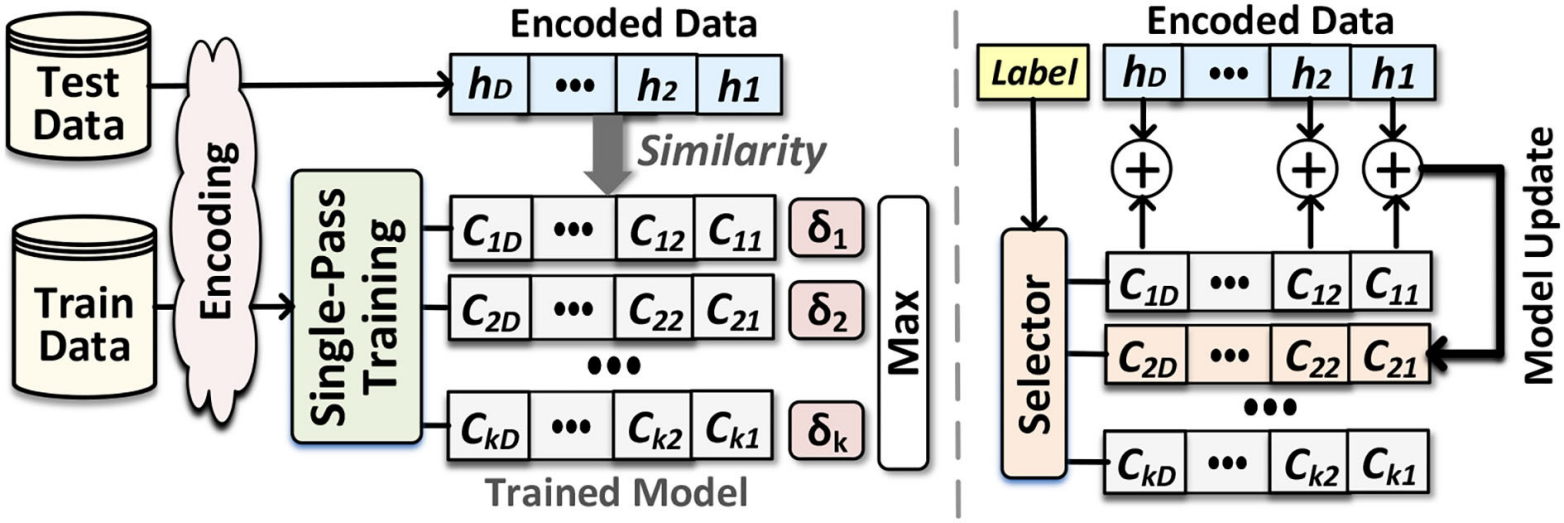
Hyperdimensional classification: Overview of EventHD for training and inference **(left)** and the routine for single-pass training **(right)**.

**Accumulative training (single-class update):** Hyperdimensional computing models receive their dataset as copies of the memory component at the point of evaluation. To find the universal property for each class in the training dataset, the trainer module linearly combines hypervectors belonging to each class, i.e., adding the hypervectors to create a single hypervector for each class. Once combining all hypervectors, we treat per-class accumulated hypervectors, called *class hypervectors*, as the learned model. Figure 3 shows HDC functionality during single-pass training. Assuming a problem with *k* classes, the model represents using: $M=\left\{\vec{C_{1}},\vec{C_{2}},\cdots \;,\vec{C_{k}}\right\}$. For example, after generating all encoding hypervector of inputs belonging to class/label *l*, the class hypervector $\vec{C^{l}}$ can be updated using: $\vec{C^{l}}=\sum_{j}^{J}\left(1-\delta \left({\vec{H}}_{j},{\vec{C}}^{l}\right)\right)\times {\vec{H}}_{j}$, where there are J inputs having label *l*. This weighted data accumulation continues for all train data available in each class. Accumulative training gives a rough estimation of a pattern of each class hypervector. However, it does not find a chance to adjust the class hypervectors for marginal predictions. This makes the HDC model sensitive to possible noise in the input data.

**Adaptive training (multi-class update):** We propose adaptive training that not only accumulates each train data with the correct class but also updates the class hypervectors with a possible marginal match. EventHD checks the similarity of each encoded query data with all class hypervectors. If an encoded query $\vec{H}$ corresponding to label *l*, the model miss-predicts it as label *l*′, the model updates 2 × *i* neighbor classes using the following equation:


(1) $$\begin{array}{l} {\vec{C}}_{l\pm i}\leftarrow {\vec{C}}_{l\pm i}+\eta_{i}\;\left(\delta_{l^{\prime }}-\delta_{l}\right)\times \vec{H}\\ {\vec{C}}_{l^{\prime }\pm i}\leftarrow {\vec{C}}_{l^{\prime }\pm i}-\eta_{i}\;\left(\delta_{l^{\prime }}-\delta_{l}\right)\times \vec{H} \end{array}$$

where $\delta_{l}=\delta (\vec{H},\vec{C_{l}})$ and $\delta_{l^{\prime }}=\delta (\vec{H},\vec{C_{l^{\prime }}})$ are the similarity of data with correct and miss-predicted classes, respectively. Unlike the accumulative training, our adaptive update provides two main features: (i) it updates multiple class hypervectors, which are centered around correct and miss-predicted classes. The neighbor class hypervector gets updated depending on its physical distance to the query (η_*i*_ sets the update ratio). This method ensures that class hypervectors have a smoother pattern of similarity; thus, a small noise in the input data cannot cause miss-prediction. (ii) Adaptive training also ensures that we update the model adaptively based on how far a train data point is miss-classified with the current model. In case of a far miss-prediction, $\delta_{l^{\prime }}>>\delta_{l}$, retraining makes major changes to the mode. While for marginal miss-prediction, $\delta_{l^{\prime }}≃\delta_{l}$, the update makes smaller changes to the model.

**Inference:** checks the similarity of each encoded test data with the class hypervector in two steps. The first step encodes the input to produce a query hypervector $\vec{H}$. Then, as Figure 3 shows, we compute the similarity (δ) of $\vec{H}$ and all class hypervectors. Query data gets the label of the class with the highest similarity.

### 4.2. Self-learning

EventHD also supports online self-learning where only a small portion of training data is labeled. EventHD exploits the HDC model transparency to improve the quality of the model. Using the techniques introduced in Imani et al. (2019a), it checks the similarity of each unlabeled data with the already trained model, obtaining the confidence level of the classification result. If the confidence level is higher than a threshold (e.g., α > 90%), EventHD updates the model by embedding encoded data into the corresponding class hypervector, as: ${\vec{C}}^{max}={\vec{C}}^{max}+\alpha \times \vec{H}$, where $\vec{H}$ is the query data and ${\vec{C}}^{max}$ is a class that has the maximum similarity with a query. EventHD exploits this same technique to update the model based on the user's feedback on the inference results. Given the absence of labels in the majority of observations, we assume that users would be willing to provide feedback when they are not satisfied and tune the confidence threshold accordingly.

## 5. Cognitive support

There is a process in the brain where the perceptual system constructs an internal representation of the world. Such an assumption has led past study in robotics and artificial intelligence to rely on the input data and their complex representation in the system for most cognitive tasks. However, recent studies in human cognition show that cognition is *enactive*: that perceiving is a way of acting, and that our perception not only depends on but is also comprised of sensorimotor knowledge (Mitrokhin et al., 2019). This makes it essential to associate the perception and the action of a model in accomplishing cognitive tasks.

### 5.1. Perception-action association

Hyperdimensional computing can naturally correlate them in high-dimensional space (Mitrokhin et al., 2019). This association enables EventHD to reason about each prediction by giving systems prior knowledge. Let us consider a system with *n*-feature as perception ($\vec{x}=\left\{f_{1},f_{2},\cdots \;,f_{n}\right\}$) and *m*-output actions ($\vec{y}=\left\{a_{1},a_{2},\cdots \;,a_{m}\right\}$) in original space. Our approach encodes both perception and action data into high-dimensional space. For perception, we exploit the proposed encoding, explained in Section 3, that preserves the spatial correlation of events. However, the output actions are often independent and do not have any spatial correlations. Therefore, our encoding method randomly generates the position hypervectors, rather than generating correlated position hypervector for a given image data. $\vec{X}=\sum_{k=1}^{m}{\vec{P}}_{k}*{\vec{L}}_{\in F}$, where $\delta \left({\vec{P}}_{i},{\vec{P}}_{i+1}\right)\sim 0$.

**Figure 4:**
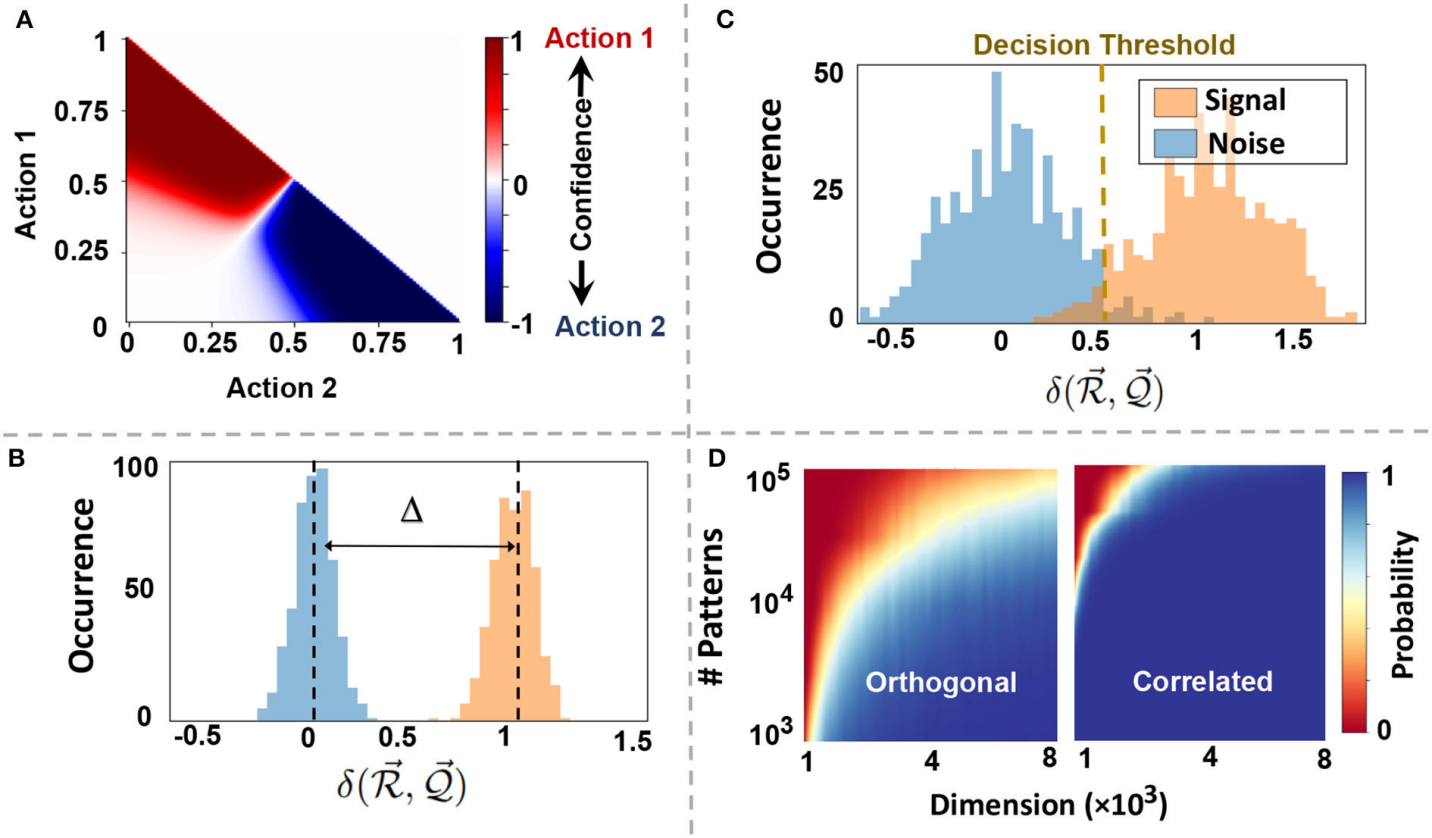
Information association and memorization: **(A)** perception-action association. Depending on the confidence of a query to the memorized perceptions measured by similarity, EventHD picks one of the actions with the highest confidence. **(B,C)** Distribution of signal and noise signal when referencing hypervector with *D* = 4*k* is storing *N* = 10^3^ and *N* = 10^4^ orthogonal patterns. When the number of patterns stored is low, like **(B)**, the distribution of the similarity of signal and that of noise are separable, implying perfect signal detection quality; when the number of patterns is high, the distributions overlap, and signal detection has less accuracy. **(D)** The capacity of reference hypervector with different dimensions storing orthogonal and correlated hypervectors. Compared to orthogonal hypervectors, correlated hypervectors require less capacity to store, resulting in higher detection probability (bluer) given fixed hyperdimension and patterns.

EventHD also encodes the output action into high-dimensional space. The action is often a single output signal. Our method linearly or non-linearly quantizes the action signal and assigns a hypervector to each quantization level, $\left\{{\vec{A}}_{1},{\vec{A}}_{2},\cdots \;,{\vec{A}}_{m}\right\}$. Our solution naturally associates each pair of perception and action by binding their corresponding hypervector. The accumulation of the bound vectors over prior observations gives native HDC-based memorization to the system: $\vec{S}=\sum_{i=1}^{n}{\vec{X}}_{i}*{\vec{A}}_{i}$. Let us assume each reference hypervector store *N* encoded perception-action hypervector: $\vec{R}={\vec{S}}_{1}+\cdots +{\vec{S}}_{N}=\sum_{j=1}^{N}{\vec{S}}_{j}$. We can predict an action for a perception ${\vec{X}}_{k}$, using:


$$\begin{array}{l} {\vec{A}}_{k}≃{\vec{X}}_{k}*\vec{R}=\left(\underset{1}{\underset{︸}{{\vec{X}}_{k}*{\vec{X}}_{k}}}\right){\vec{A}}_{k}+\overset{N}{\underset{i=1}{\sum }}\left(\underset{\text{Noise}}{\underset{︸}{{\vec{X}}_{k}*{\vec{X}}_{i}}}\right){\vec{A}}_{i} \end{array}$$

where ${\vec{A}}_{k}$ is an interpolation between all actions that their perceptions have high similarity to ${\vec{X}}_{k}$. Figure 4A shows EventHD selecting between two discrete actions. Depending on the confidence, i.e., the similarity of a query to memorized perceptions, EventHD picks one of the actions. In continuous space, the selection translates to interpolation between the actions, depending on the perceptions similarity in HDC space.

### 5.2. Memorization in perception-action space

In HDC, bundling acts as a memory, storing the information of multiple encoded hypervectors into a single reference hypervector. EventHD exploits bundling to memorize the associated perception-action, $\vec{R}=\overset{N}{\underset{j=1}{\sum }}{\vec{S}}_{j}$. The reference hypervector has limited capacity and, thus, cannot store the information of unlimited encoded data. The capacity depends on the dimensionality and the orthogonality of the encoded hypervectors. For a given query data, EventHD can refer to memory in order to retrieve the system's prior knowledge. For example, let us assume *q* is a perception with $\vec{Q}$ being its encoded data. EventHD can retrieve information about possible actions by checking the similarity of the query with the reference model:


$$\begin{array}{l} \delta \left(\vec{R},\vec{Q}\right)=\underset{\text{Signal}}{\underset{︸}{\delta \left({\vec{S}}_{\lambda },\vec{Q}\right)}}+\underset{\text{Noise}}{\underset{︸}{\overset{N}{\underset{i=1,i\neq \lambda }{\sum }}\delta \left({\vec{S}}_{i},\vec{Q}\right)}} \end{array}$$

If ${\vec{P}}_{\lambda }=\vec{Q}$ for some λ, the output of the function is going to be ${\vec{A}}_{\lambda }$. For reference patterns that do not match with the query, the similarity is nearly zero, $\delta \left({\vec{S}}_{i},\vec{Q}\right)≃0$. Thus, we can check the existence of a query $\vec{Q}$ in $\vec{R}$ using the following criteria: $\delta \left(\vec{R},\vec{Q}\right)/D>T$, where *T* is a threshold and $\delta \left(\vec{R},\vec{Q}\right)/D$ is called the *decision score*.

Figures 4B,C show the normalized distribution of signal and noise in EventHD information retrieval (using *D* = 10*k*). These Gaussian distributions determine the capacity of each reference hypervector in memorizing the information. As our mathematical model indicated, the noise is getting a wider distribution when increasing the number of patterns stored in $\vec{R}$. When the noise overlaps with the signal, there is no threshold *T* that can separate noise, thus resulting in information loss (Figure 4C). Figure 4D shows the capacity of reference hypervector with *D* dimensionality in storing *N* nearly orthogonal and correlative patterns. Our evaluation shows that the capacity of the reference hypervector increases with dimensionality. For example, EventHD with *D* = 4*k* can stored *N* = 10^3^ (*N* = 10^4^) orthogonal patterns with less than 0.5% (10%) information loss. Note that, in practice, the reference hypervector has a much higher capacity as EventHD encoder keeps the correlation between input signals. As Figure 4D shows, a reference hypervector provides significantly higher capacity when the EventHD encoder preserves correlation in high-dimensional space. For a more in-depth analysis of the memory capacity, readers are referred to Frady et al. (2018).

### 5.3. Other applications: Beyond memorization

EventHD similarity search on the memorized model gives us an estimation of the output action. EventHD uses this prediction as prior knowledge to trust the prediction. If the prediction is relatively far from the memorized action, EventHD gives very low confidence to that prediction. This approach enables us to reason about each prediction and potentially provide a more explainable learning solution.

## 6. Evaluation

### 6.1. Experimental setup

We implement EventHD using software, hardware, and system implementation. In software, we verified EventHD training and testing using a C++ implementation. For hardware, we design the EventHD functionality using Verilog and synthesize it using Xilinx Vivado Design Suite (Feist, 2012). The synthesis code has been implemented on the Kintex-7 FPGA KC705 Evaluation Kit. We ensure our efficiency is higher than the automated FPGA implementation at (Salamat et al., 2019).

We evaluate EventHD accuracy and efficiency on two Datasets: the Neuromorphic MNIST (N-MNIST) and the Multi-Vehicle Stereo Event Camera (MVSEC) dataset. N-MNIST is an event-based version of the MNIST dataset, containing event-stream recordings of the 60,000 training digits and 10,000 testing digits. The MVSEC dataset collects event-based DVS cameras on the self-driving car day and night (Zhu et al., 2018; Mitrokhin et al., 2019). This dataset is designed for regression tasks to predict the car velocity based on DVS data. The experiments correspond to mDAVIS-346B cameras with 346×260 pixel resolution. To find ground truth velocity values, the car is equipped with IMUs and GPS sensors. The evaluation is performed for five activities, two recorded during the day and three in the evening/night. The results are reported using two metrics: Average Relative Pose Error (ARPE) and Average End-point Error (AEE). ARPE shows the average angular error between translational vectors while ignoring the scale (Mitrokhin et al., 2019), while AEE shows the absolute error in 2D linear space. Similar to other error metrics, the lower ARPE and AEEE indicate higher quality of learning. All results are reported for MVSEC data unless they are stated differently. We use a simple DNN with one 512-neuron hidden layer as our baseline from conventional neural networks. EventHD is configured to have a hyperdimension of *D* = 4, 000, a window size of *k* = 5 for positional, and a time window size of *t* = 50(*ms*) across all experiments, as it leads to the best average performance.

### 6.2. EventHD accuracy

**Figure 5:**
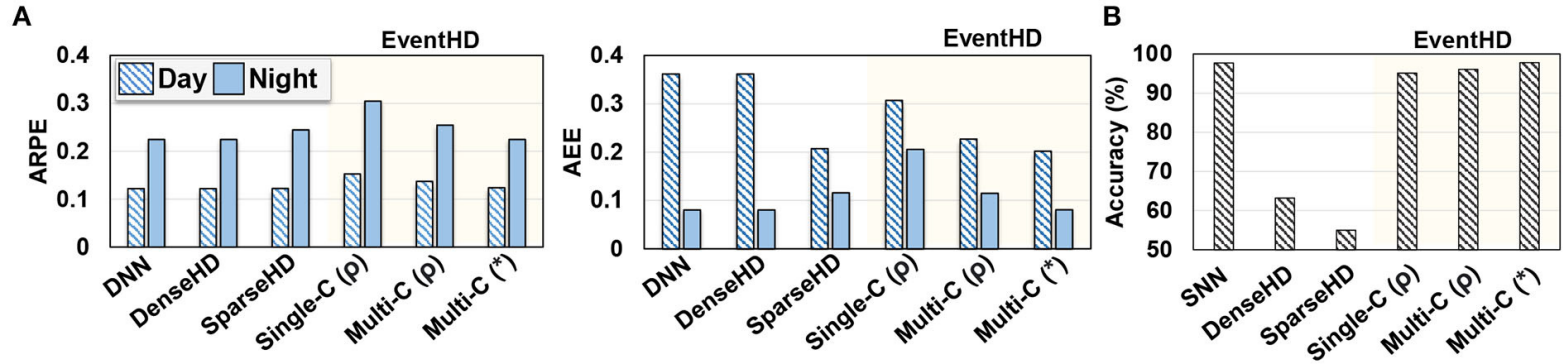
EventHD quality of learning over MVSEC and N-MNIST datasets **(A,B)**. The results are compared to the state-of-the-art HDC-based approach. For DNN, SNN, SparseHD, and DenseHD, we use their original implementation and allow preprocessing as needed. For EventHD, we report the results for single-class (Single-C) and multi-class (Mult-C) updates. For multi-class, we also report results for permutation-based temporal encoding (ρ) and association-based temporal encoding (*). Evaluations for MVSEC are measured by Average Relative Pose Error (ARPE) and Average End-point Error (AEE), and that for N-MNIST is classification. accuracy. EventHD provides comparable or better quality of learning compared to state-of-the-art solutions.

Figure 5A compares EventHD quality of learning over classification task, using both day and night data. We compare EventHD with state-of-the-art HDC methods working on event-based sensors: DNN, Dense HDC (DenseHD) (Mitrokhin et al., 2019), and Sparse HDC (SparseHD) (Hersche et al., 2020). All three baseline approaches operate over six extracted features by the preprocessing method. In contrast, EventHD is an end-to-end framework that directly operates over raw neuromorphic data. Note that other algorithms, i.e., DNN, DenseHD, and SparseHD, provide close to a random prediction when processing the raw neuromorphic data. For EventHD, we report the results for single-class (Single-C) and multi-class (Mult-C) updates using both ARPE and AEE metrics. Our evaluation shows that EventHD using both accuracy metrics provides comparable or better quality of learning compared to the state-of-the-art solutions. For example, EventHD ARPE (AEE) error metric is, on average, 0.1% and 4.8% (37.0% and 14.1%) lower than DenseHD and SparseHD, respectively. These metrics indicate EventHD higher quality of learning. Note that EventHD efficiency and robustness are significantly higher than all baseline methods due to eliminating costly preprocessing (detailed evaluation in Section 6.3).

Figure 5B also evaluates the EventHD quality of learning on the N-MNIST dataset. The results are compared to SNN and HDC-based neuromorphic approaches. Unlike EventHD which operates over raw neuromorphic data, SparseHD and DenseHD rely on preprocessing algorithms to extract spatial-temporal information. Our evaluation shows that EventHD provides significantly higher classification accuracy than existing HDC-based algorithms, i.e., SparseHD and DenseHD.

**Temporal encoding:** Figure 5 also compares EventHD accuracy using permutation-based (ρ) and association-based (*) temporal encoding. Our evaluation shows that the association-based encoding provides a lower error rate by enabling a notion of continuous-time dynamic, while permutation-based encoding only preserves the orders of events. For example, EventHD using association-based provides 10.2% (17.2%) lower ARPE (AEE) compared to a permutation-based solution on the MVSEC dataset.

**Figure 6:**
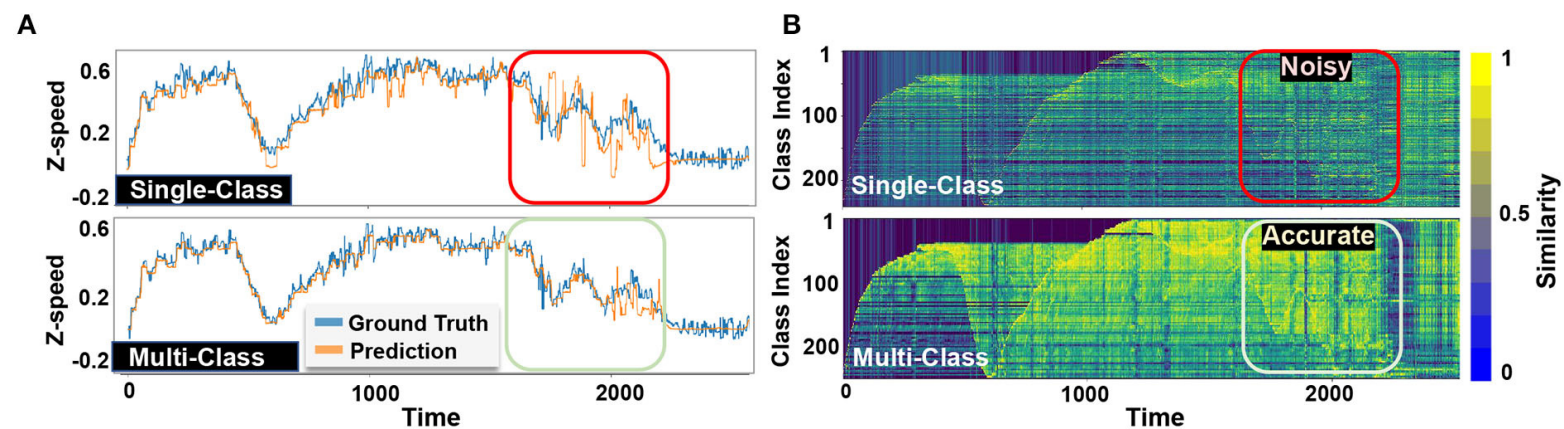
Visualization of classification results of EventHD prediction on single-class and multi-class update configurations for z-axis linear speed of MVSEC outdoor night 1: The model is trained on the first 1,000 ground truth samples and then used to predict up to 2,500 samples as indicated by the time axis. **(A)** Visualize EventHD final prediction for linear speed along z-axis compoared to ground truth and **(B)** Display the similarity between the query and each class hypervectors.

**Single vs. multi-class:** Figure 6 visually compares EventHD classification accuracy in two configurations over the MVSEC dataset: a single-class and a multi-class update. In both configurations, we show the final prediction (Figure 6A) and the similarity of a query with different class hypervectors (Figure 6B). EventHD with a single-class update creates a weak learning model with high sensitivity to noise and variation in the input data. Therefore, during inference, it may deviate toward the wrong class. However, our multi-class update solution keeps the correlation between the predicted speeds and strength of the class hypervectors, thus providing higher learning accuracy. The box in Figure 6 clearly shows the capability of EventHD multi-class update to strengthen the signal in related class hypervectors and provide a higher quality of prediction.

**Figure 7:**
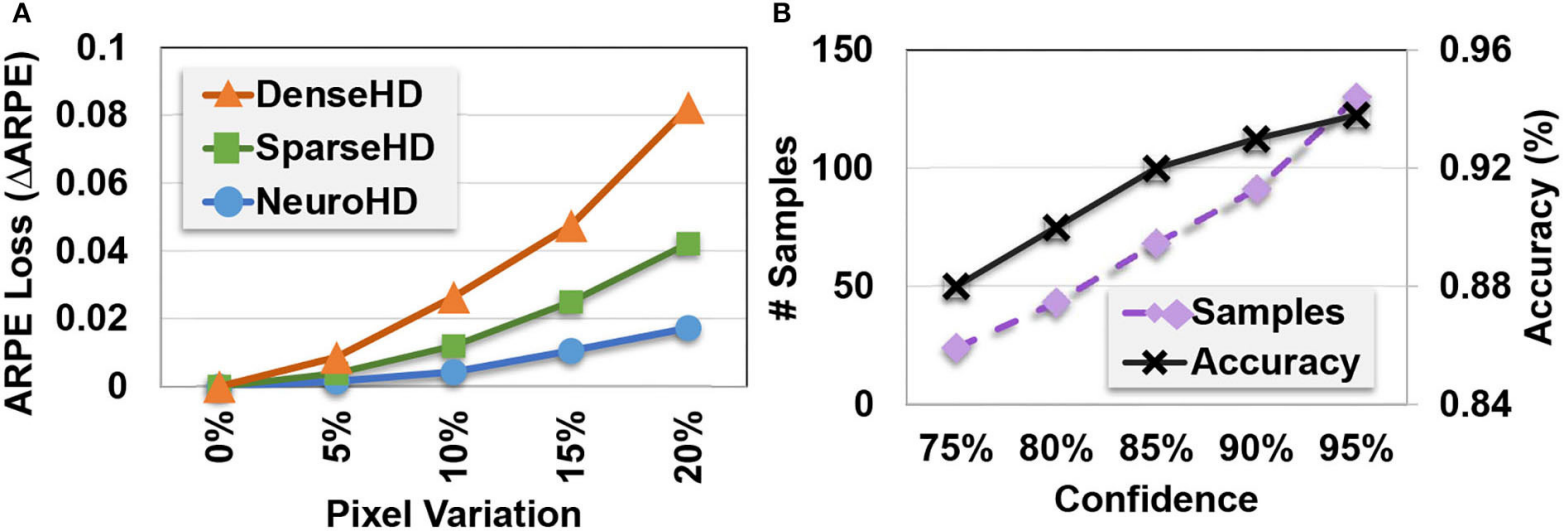
EventHD robustness and self-learning capability: **(A)** Robustness to EventHD and other HDC-based algorithms to pixel variation and **(B)** EventHD self-learning over unlabeled data (semi-supervised).

**Robustness to variation:** Unlike prior HDC-based approaches that do not keep the correlation, EventHD encoding is asynchronous, thus preserving both temporal and spatial correlation over event-based data. We perform an experiment to show EventHD capability to respond to noisy data. Figure 7A shows EventHD and HDC quality of learning when the activated events in each timestamp are randomly shifted in an arbitrary direction. Our evaluation shows that EventHD is highly robust against such possible variational data, as it provides the maximum accuracy even using a 5% shift. In contrast, the state-of-the-art HDC solutions do not keep the correlation between neighbor pixels (spatial correlation). Therefore, a single shift operation can generate a signal which is entirely orthogonal to the non-shifted version. As Figure 7A shows, this makes the existing HDC solutions, DenseHD and SparseHD, very sensitive to possible noise or variation in the input signal.

**Self-learning:** Figure 7B shows EventHD classification accuracy during the self-learning iterations. The results are reported when EventHD has been trained, supervised over 10% of train data, and unsupervised over the other 90%. Our evaluation shows that EventHD can adaptively improve classification accuracy during self-learning. This advantage comes from the EventHD capability of computing confidence for each prediction. Therefore, EventHD trusts data with high confidence for model updates while ignoring low confidential data. On another side, a higher confidence threshold increases the required train samples to converge to maximum accuracy.

### 6.3. EventHD efficiency and robustness

We compare EventHD efficiency and robustness to state-of-the-art HDC solutions. The results are for the total processing time, including both preprocessing and learning. The existing HDC solutions use image-to-time transformation as a preprocessing step for feature extraction from the event-based information. The feature extraction result is only a few feature data (i.e., six features in our example). The preprocessing makes the learning task very simple such that even a simple learning solution, e.g., linear regression or perceptron, can provide acceptable accuracy. Due to the complexity of the preprocessing step, its cost eliminates the effectiveness of HDC in enhancing system efficiency. In contrast, EventHD is an end-to-end solution, directly operating over the raw data received by the event-based camera. Our solution eliminates the costly preprocessing step by enabling HDC encoding to preserve both the temporal and spatial locality of the raw data. This improves not only EventHD computation efficiency but also provides significant computational robustness.

**Figure 8:**
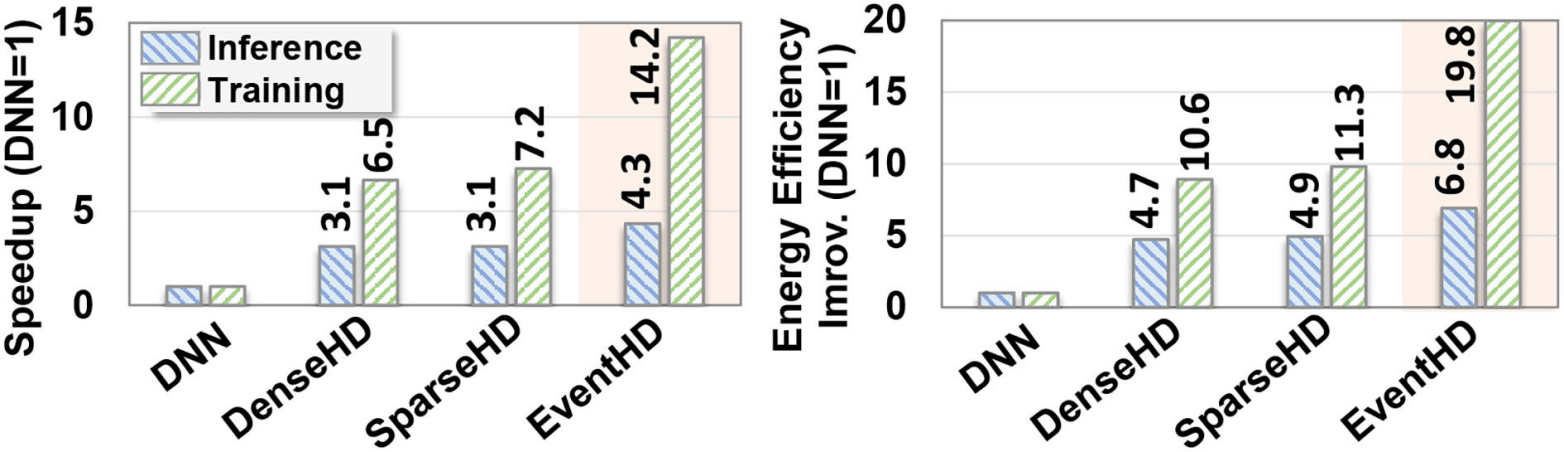
Efficiency analysis: Comparison of EventHD performance speedup and energy efficiency with state-of-the-art algorithms on the FPGA platform. The results are reported for both training and inference phases and is normalized relative to DNN performance and energy efficiency. During training (inference), EventHD achieves, on average, 10.6× (14.2×) faster and 16.3× (19.8×) more energy-efficient computation as compared to FPGA-based DNN implementation, respectively.

**Efficiency:** Figure 8 compares EventHD computation efficiency with the existing HDC solutions running on FPGA. The results are reported for both training and inference phases. For DNN, we used DNNWeaver V2.0 (Sharma et al., 2016) for the inference and FPDeep (Geng et al., 2018) for training implementation on a single FPGA device. For DenseHD and SparseHD, we use the F5-HD (Salamat et al., 2019) framework for FPGA implementation. All FPGA implementations are optimized to maximize performance by utilizing FPGA resources. All results, in Figure 8, are relative to DNN performance and energy efficiency. During training, EventHD achieves, on average, 10.6× faster and 16.3× more energy-efficient computation as compared to FPGA-based DNN implementation, respectively. The high efficiency of EventHD in training comes from EventHD capability in (i) creating an initial model that significantly lowers the number of required retraining iterations and (ii) eliminating the costly gradient for the model update. This results in higher EventHD efficiency, even in terms of a single training iteration. In inference, EventHD provides 4.3× faster and 6.8× higher energy efficiency as compared to FPGA-based DNN implementation. As compared to SparseHD (DNN), EventHD provides 1.9× and 2.1× (14.2× and 19.8×) faster and more energy-efficient training. The main computation efficiency comes from eliminating the costly preprocessing step and replacing it with HDC encoding.

**Robustness:** The noise in today's technology is coming from multiple sources. Unfortunately, the existing data representation has very low robustness to noise in hardware. An error bit on the exponents or Most Significant Bits (MSBs) result in a major change in the weight value, while an error in the Least Significant Bits (LSBs) adds minor changes to the computation. The randomness of the noise makes traditional data representations vulnerable to an error on the hardware. One of the main advantages of EventHD is its high robustness to noise and failure. In EventHD, hypervectors are random and holographic with i.i.d. components. Each hypervector stores the information across all its components so that no component is more responsible for storing any piece of information than another. This makes a hypervector robust against errors in its components. EventHD robustness depends on the hypervector dimensionality that determines the hypervector capacity and redundancy. Table 1 compares EventHD robustness with the existing HDC and learning solutions operating the preprocessing or the entire learning task over the original data representation. The results indicate that EventHD quality of learning is almost constant, even using 5% noise. In contrast, even a small amount of error on an existing solution can result in significant quality loss. For example, under 20% random noise, EventHD using *D* = 4*k* provides 17.8% and 14.7% higher accuracy than DenseHD and SparseHD, respectively. Note that EventHD robustness increases with its dimensionality (as shown in Table 1). However, higher dimensionality results in lower computation efficiency.

**Table 1:** Robustness analysis of different learning algorithms to hardware error rate.

**Error rate**	**1%**	**2%**	**5%**	**10%**	**15%**	**20%**
DNN	0.7%	1.9%	3.7%	11.5%	21.3%	38.6%
Dense HDC (Mitrokhin et al., 2019)	0.2%	1.0%	1.7%	6.8%	14.2%	18.3%
Sparse HDC (Hersche et al., 2020)	0.2%	0.9%	1.9%	6.3%	12.8%	21.4%
EventHD (*D* = 4 k)	0.0%	0.0%	0.2%	0.8%	1.2%	3.6%
EventHD (*D* = 8 k)	0.0%	0.0%	0.1%	0.6%	0.8%	2.4%

For each experiment, random bit flips of the model parameters are applied according to the error rates, and the average absolute accuracy drop over N-MNIST classification is reported.

## 7. Related study

In recent years, HDC has been employed in a range of applications, such as text classification (Kanerva et al., 2000), activity recognition (Kim et al., 2018), biomedical signal processing (Rahimi et al., 2018), multimodal sensor fusion (Räsänen and Saarinen, 2015), and distributed sensors (Kleyko and Osipov, 2014; Kleyko et al., 2018). A key HDC advantage is its training capability in a single pass, where object categories are learned as opposed to many iterations. HDC has achieved comparable to higher accuracy compared to state-of-the-art machine learning models with lower execution energy. Much research also exploits the memory-centric nature of HDC to design in-memory acceleration platforms (Li et al., 2016; Halawani et al., 2021a,b) However, existing HDC algorithms are often ineffective in encoding complex image data or keeping a notion of continuous-time. In contrast, we propose a novel method to preserve spatial-temporal correction, where spatial encoding keeps the correction of events in 2D space while temporal encoding defines correlation in a continuous-time dynamic.

In the context of neuromorphic computing, study in Mitrokhin et al. (2019) and Hersche et al. (2020) exploited HDC mathematics to learn from event-based neuromorphic sensors. However, these designs have the following challenges: (i) rely on the expensive preprocessing step to extract information from event-based sensors, (ii) lack computational robustness, as the preprocessing step operates over original data with high sensitivity to noise, and (iii) require heterogeneous data processing and non-uniform data flow to accelerate HDC and preprocessing step. In contrast, to the best of our knowledge, EventHD is the first HDC-based solution that directly operates over raw data received by the event-based sensors. EventHD not only enhances the learning efficiency but also results in a significantly higher computational robustness to noise in input or underlying hardware.

## 8. Conclusion and future study

In this article, we present EventHD, an end-to-end framework based on hyperdimensional computing for robust, efficient learning from neuromorphic sensors. EventHD proposes a novel encoding scheme to map event-based neuromorphic data into high-dimensional space while preserving spatial and temporal correlation. Then, EventHD exploits HDC mathematics to support learning and cognitive tasks over encoded data by inherently exploiting the associating and memorizing capabilities. Finally, we introduce a scalable learning framework to distribute EventHD computation over devices in IoT networks.

Our future study will exploit EventHD encoding to enhance current spiking neural networks (SNNs). Particularly, SNN and HDC have shown promising results in enabling efficient and robust cognitive learning. However, despite their success, these two brain-inspired models are complementary. While SNN mimics the physical properties of the brain, HDC models the human brain on a more abstract and functional level. Our goal is to exploit EventHD encoding to fundamentally combine SNN and HDC to design a scalable and strong cognitive learning system that better mimics brain functionality.

## Data availability statement

The original contributions presented in the study are included in the article/supplementary material, further inquiries can be directed to the corresponding author/s.

## Author contributions

ZZ and MI conceived the research. ZZ, HA, YK, MN, NS, and MI conducted the research and analyzed the data. ZZ, HA, YK, and MI wrote the manuscript. All authors reviewed the manuscript and agreed on the contents of the manuscript.

## Funding

This study received funding from National Science Foundation (NSF) #2127780 and #2019511, Semiconductor Research Corporation (SRC) Task No. 2988.001, Department of the Navy, Office of Naval Research, grant #N00014-21-1-2225 and #N00014-22-1-2067, Air Force Office of Scientific Research, grant #22RT0060, the Louisiana Board of Regents Support Fund #LEQSF(2020-23)-RD-A-26, and generous gifts from Cisco. The funders were not involved in the study design, collection, analysis, interpretation of data, the writing of this article, and the decision to submit it for publication.

## Conflict of interest

NS was employed by the company Intal Labs. The remaining authors declare that the research was conducted in the absence of any commercial or financial relationships that could be construed as a potential conflict of interest.

## Publisher's note

All claims expressed in this article are solely those of the authors and do not necessarily represent those of their affiliated organizations, or those of the publisher, the editors and the reviewers. Any product that may be evaluated in this article, or claim that may be made by its manufacturer, is not guaranteed or endorsed by the publisher.
